# Nanoporous UHMWPE Membrane Separators for Safer and High‐Power‐Density Rechargeable Batteries

**DOI:** 10.1002/gch2.201700020

**Published:** 2017-05-11

**Authors:** Runlai Li, Ping Gao

**Affiliations:** ^1^ Department of Chemical and Biomolecular Engineering The Hong Kong University of Science and Technology Clear Water Bay Kowloon Hong Kong

**Keywords:** LIB separators, nanoporous membranes, self‐reinforced composite, shape retention, UHMWPE

## Abstract

Battery safety has been of critical concerns and there are renewed interest in developing safer membranes for enhancing the inherent safety of lithium ion batteries. In this paper, the synthesis of a robust and safer self‐reinforced composite ultrahigh molecular weight polyethylene (UHMWPE) membrane is described. The self‐reinforced composite membrane consists of ≈200 nm nanopores homogeneously embedded inside interpenetrating nanofibrillar “shish kebab” networks. It performs thermal fuse function by selectively melting its kebab crystals while the elongated shish fibrillary backbones remain intact. Simulated thermal fuse function tests show that the newly prepared separator displays a 300% increase in tensile strength (550 MPa), 300% increase in puncture resistance (1.5 N μm^−1^), as well as an 18 000 times increase in impedance when lateral dimensions are kept constant. Cells prepared using the UHMWPE separators also exhibit a 10% higher energy density and better cyclability than those using commercial separators. Hence, the newly prepared ultrathin and dimensionally stable membrane will enhance the safety protections for rechargeable batteries with low impedance for high energy and power density.

## 1 Introduction

Rechargeable lithium ion batteries (LIBs) are widely used in portable consumer electronics, electrical vehicles, and increasingly explored for use in the energy storage market to supplement environmentally friendly power sources such as wind and solar energy.^1^, ^2^, ^3^, ^4^ The construction of an LIB consists of an anode, a cathode, and a separator. The separator acts to prevent the direct contact between the two electrodes while providing conductive paths for lithium ions transport by serving as the electrolyte reservoir. Thus it is the most critical component in determining the inherent stability and safety of the battery.^5^ Safety of LIBs has been a serious concern notably with the recent Samsung Note 7 mobile phone explosions and the Boeing 747‐400 cargo plane that caught on fire.^6^, ^7^

Currently, all commercial spiral wound LIBs use polyolefin microporous separators due to their safety, chemical resistance, and low cost. These membranes all exhibit shutdown functions and can provide the required performance and inherent safety features.^8^, ^9^ For example, the thermal fuse function of a commercially available LIB separator made of poly(propylene)/polyethylene/poly(propylene) sandwich structures is due to the sacrificial melting of the intermediate polyethylene layer.^10^ The porous polyethylene (PE) film has a lower melting temperature than poly(propylene) (PP) and helps to shut down the circuit when the internal temperature rises above its melting temperature. In the meantime, the outer PP layers are still in the solid state and hence provides the mechanical integrity to prevent direct electrode contact. However, the major issues with PP separators are their slit‐like pore structures and their poor tear resistance.^11^ Any punctures produced on the membrane are likely to induce a catastrophic failure of the entire system. PE membranes, particularly ultrahigh molecular weight polyethylene (UHMWPE) membranes, are more desirable due to their higher toughness, lower shutdown temperature, and lower glass transition temperature favoring low temperature performances.^12^ Incorporating UHMWPE in high density polyethylene membranes improves both the tensile strength and puncture resistance of PE separators.^13^ However, UHMWPE concentrations above 10 wt% have caused significant processability issues due to the higher solution viscosities.

It is believed that high melting temperature polymer membranes may overcome the melt down issues pertaining to polyolefin separators. For example, a sandwich of PVdF/PMIA/PVdF with high electrolyte affinity and shape stability has been reported.^14^ However, these membranes are much more expansive and show less than 10% of the mechanical strengths (≈10 MPa) of polyolefin separators. Besides, these materials will eventually be melt down due to their low melt viscosity.

Herein we highlight the design of a self‐reinforced composite UHMWPE membrane separator that improves the inherent safety of LIBs without sacrificing high‐energy and high‐power densities. This design takes the advantage of high‐mechanical toughness and super‐high melt viscosity of UHMWPE materials.

## 2 Results and Discussions

### 2.1 Morphology of UHMWPE Membranes

**Figure 1:**
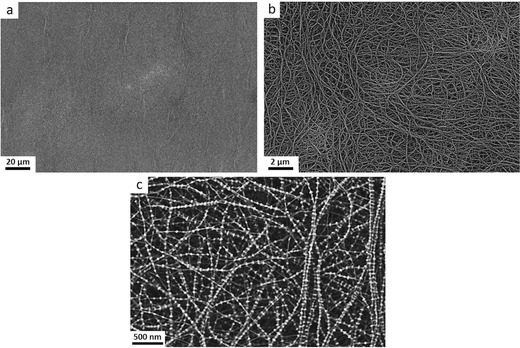
Typical SEM images of biaxially oriented nanoporous UHMWPE membrane: a–c) SEM photograph of P‐class membrane surface.

**Figure** **1** shows the SEM images of the UHMWPE membrane separator (P‐class) surfaces at different magnifications under a low‐voltage scanning electron microscope (LV‐SEM). The lower magnification image in Figure 1a,b shows that the newly prepared porous membrane is highly homogeneous and consists of interpenetrating fibrous network structures. The higher magnification image in Figure 1c reveals that the interpenetrating nanofibrillar networks are the frameworks for the confinement of the uniformly distributed ≈200 nm diameter pores. The nanofibrils are strings of pearl necklaces of “shish‐kebab” crystals randomly aligned in the plane surface. The cores of the necklaces are made of extended chain shish crystals with diameters ≈16 nm and the periodically (spacing ≈100 nm) attached beads with thicknesses ≈32 nm and widths ≈45 nm are the folded chain kebab crystals.^15^, ^16^, ^17^

Polymer melts and solutions undergo flow‐induced transitions into shish‐kebab self‐reinforced composite crystalline structures due to coil‐stretch transformations proposed by de Gennes.^18^ Very few studies are available on the self‐reinforced composite shish kebab crystalline structures in porous films or fibers. Mechanistically, these kebab crystals are due to the annealing induced partial melting and recrystallization of the imperfectly aligned shish crystals where the unmelted shish crystals act as the nucleation sites for these kebabs.^19^ This process is analogous to that for the formation of self‐reinforced composite of compacted UHMWPE gel‐spun fibers.^20^

The volumetric porosity of the newly prepared UHMWPE membrane was characterized by liquid absorption technique. Weight uptake of mineral oil at room temperature was performed and the porosity was estimated according to Equation (1)

(1) $$\rho \left(\%\right)=\left(\frac{W-W_{0}}{\rho_{L}V_{0}}\right)\;\;\times \;100$$

where *W*, *W*_0_ are the weights of the separator membrane before and after immersion in liquid mineral oil, ρ_*L*_ the density of mineral oil, and *V*_0_ the geometric volume of the separator membrane, respectively.^21^ This gives a volumetric porosity of 78.3%. This is about 56% higher than typical commercial polyolefin membranes and hence the P‐class membrane developed in this study is expected to exhibit higher electrolyte uptake and lower impedance.^10^

**Figure 2:**
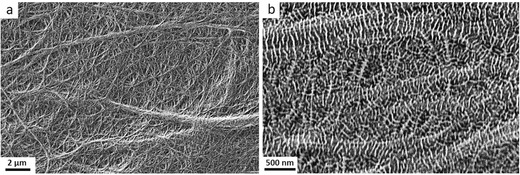
D‐class membrane: a,b) SEM photograph of D‐class membrane surface.

Simulated thermal fuse functionality of these P‐class membranes were carried out by subjecting these membranes to an annealing temperature of 145 °C for 7 min under lateral dimension constraints. This temperature is above the melting temperatures of the kebab crystals but lower than that of the shish‐crystals determined under differential thermal calorimetry (DSC). The membranes after thermal annealing are categorized as D‐class, and their surface microstructures are depicted in **Figure** **2**. The lower magnification SEM image in Figure 2a shows that the D‐class membrane retains the shish‐kebab self‐reinforced composite and interpenetrating fibrous network structure reminiscent of that of the P‐class. In the meantime, the number density and widths of the epitaxial kebabs increased significantly. As can be seen from the magnified image in Figure 2b, the inter kebab distance decreased by about 40% to 60 nm. In the meantime, there is a small decrease in kebab thickness to ≈25 nm. The widths of the kebabs doubled to ≈92 nm. The shish diameter also becomes twice as thick to ≈30 nm. These microstructural changes suggest two different mechanisms were at play for the pore shutdown process. The first is the melting and recrystallization of kebabs, and the other is the thickness contraction that leads to the densification of films. Physical constraints of lateral dimensions during thermal annealing ensures only thickness contraction was possible during the pore closure process. The film thickness decreased from ≈6 μm (P‐class) to ≈2 μm (D‐class) after thermal annealing.

**Figure 3:**
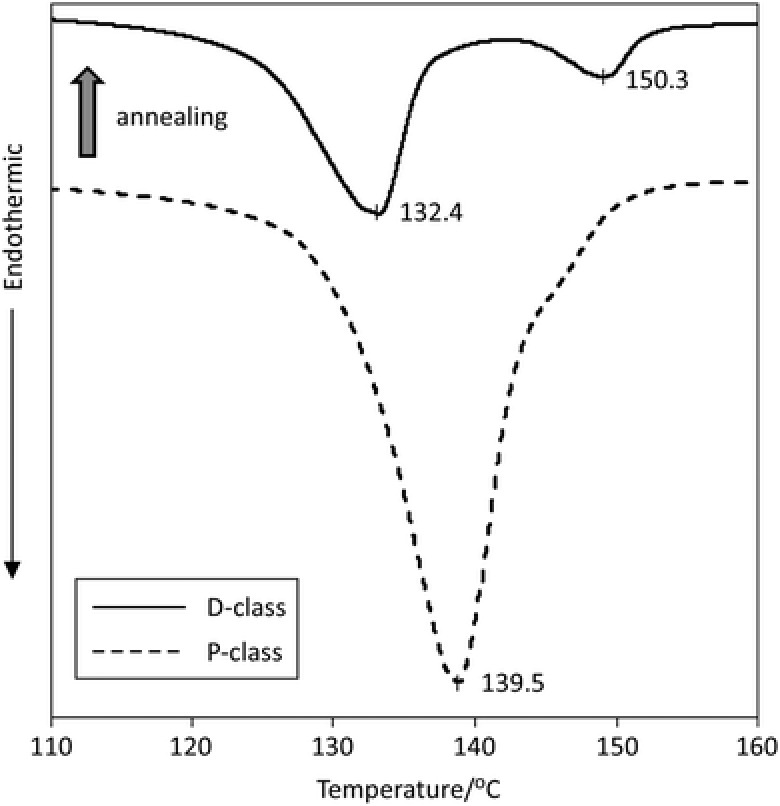
DSC heat flow rate curve of membranes before (P‐class) and after (D‐class) pore closure.

In order to elucidate these morphological changes, thermal transition behavior of the membranes before and after pore closure were characterized under DSC at a standard heating/cooling rates of 10 °C min^−1^. All measurements were performed on samples confined by compaction of aluminum flat pans. The plots depicted in **Figure** **3** show the heating endotherms for the P‐class (dashed line) and D‐class (solid line) membranes, respectively. The P‐class exhibits a broad melt transition endotherm with a melting temperature of 139 °C and a broad shoulder at ≈145 °C. Integration of the melt transition endotherm gives a crystallinity of ≈86% by assuming a heat of fusion of 293.6 J g^−1^ for polyethylene single crystals.^22^

The broad melting transition depicted here is consistent with that expected from the SEM images in Figure 1. The folded‐chain kebab crystals display lower melting temperatures than the equilibrium melting temperature of 143 °C for polyethylene and the extended‐chain shish crystals may superheat and are expected to display higher melt transition temperature.^19^ The D‐class membranes (solid line) show two distinct melting peaks: the first melting peak is at ≈132 °C, and a second at ≈150 °C. The lower melting peak is now lower than that of the P‐class (dashed line), but the higher one is higher and more pronounced.

The observed decrease in the lower melting temperature of the endotherm plot of D‐class membrane is consistent with the fact that the kebab thickness in the D‐class membranes is ≈21% thinner than those in the P‐class (25 vs 32 nm). The increase in melting temperature of the shish crystals is also consistent with that expected from the doubling shish diameters in Figure 2b. As the shish diameter increases, the number of aligned chains in each fibrillar shish crystal increases and hence resulting a stronger superheating effect.^23^ On the other hand, the D‐class membrane exhibits a crystallinity of ≈52% which is 40% lower than that the P‐class.

Both the lower crystallinity and thinner kebab dimension support our hypothesis that the pore closure is mainly due to the melting of the kebab crystals. Kebab crystals in the P‐class membrane become molten during thermal annealing at 145 °C. Some of these molten polymer chains are able to form epitaxial kebab crystals on the shish‐backbones upon cooling and some will remain amorphous. This is due to the drastically reduced chain mobility in molten high entanglement density UHMWPE. The thickening of the shish‐crystals is most likely a result of the densification of the membrane in the thickness direction during thermal annealing.

### 2.2 Mechanical and Electrochemical Characterizations

**Figure 4:**
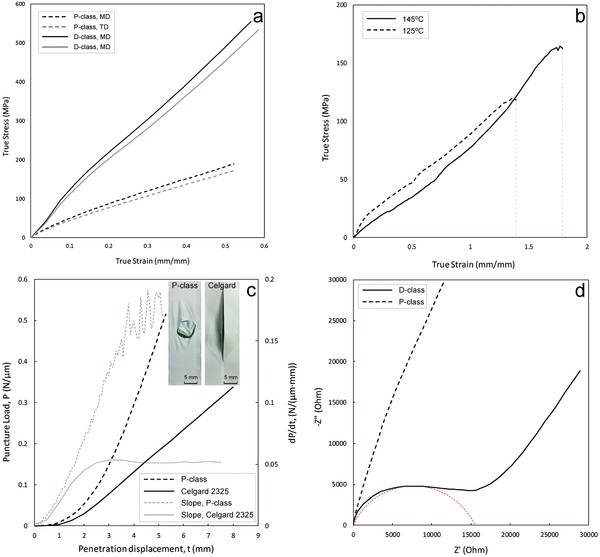
a) True stress versus Hencky strain curves of membranes before (P‐class) and after (D‐class) thermal treatment. b) True stress–strain curves of P‐class membranes tested at 125 and 145 °C (pore‐closure temperature). c) Force‐penetration displacement curves of P‐class and Celgard 2325 for puncture resistance test. The inserts are the images of the samples after puncture fracture. d) Nyquist plots of membranes before (P‐class) and after (D‐class) pore closure sandwiched in between blocking electrodes.

Safety of LIBs includes tensile strengths to withstand spiral winding load, puncture strength to mitigate particle pultrusion, dimensional stability and mechanical integrity in case of a thermal runaway reaction, as well as a rapid impedance increase for electrical circuit shutdown capability. Summary of the safety performance of these membranes is presented in **Figure** **4**.

Figure 4a shows the tensile stress–strain curves of the P‐ and D‐class membranes at the ambient temperatures. In order to characterize the drawing sequence effect on the membrane's mechanical performance, measurements were carried out both in the machine direction (first drawing direction) and in the transverse direction (second drawing direction). The two dash‐lines represent the data measured on the P‐class and the two solid lines on the D‐class. Clearly, the maximum differences in tensile stresses between drawing directions are less than 5% and it may be safely assumed that the molecules in the plane direction have no preferred molecular orientations.

The tensile strengths of the separator P‐class membrane (dashed lines) in Figure 4a are ≈180 MPa. This similar to that of Celgard membranes measured in the machine direction, but more than 1100% higher than those measured in the transverse direction of Celgard membranes. It is 800% higher than the Celgard membranes measured at 45° to the machine direction.^24^

As the battery may become overheated during charging, dimensional stability and mechanical integrity are critical to mitigate electrode contacts. We observed that the lateral dimensions of the UHMWPE separators are readily constrained via clamping between two metal plates suggesting high dimensional stabilities against abuse temperature effects. Plots in Figure 4b show the tensile behavior of the membrane at 125 °C (dashed line, before pore shutdown) and 145 °C (solid line, after pore shutdown) under lateral dimensional constraints via film folding on the sides transverse to the displacement direction. Clearly, the UHMWPE membranes are highly ductile extremely strong. Elongation and tensile stress prior to fracture reached 350% (500% at 145 °C) and 130 MPa, respectively. Lateral dimension contraction was readily suppressed by folding the membranes one the sides during tensile testing. The data presented in Figure 4b had been subtracted from those folded edges measured independently. (See movie for tensile draw at 125 and 145 °C, respectively, in Videos S1 and S2, Supporting Information)

In addition to tensile resilience, membranes must possess sufficient puncture resistances to mitigate potential short circuiting of LIBs due to piercing failures induced by micrometer‐sized particles in the system. Two sources of micrometer‐sized particles are identified: one kind is the loosened catalytic particles falling from the electrodes during spiral winding process and another the lithium dendrites formed after prolonged period of charge–discharge cycles. The size of these particles limits the minimum separator thickness for safety requirement.^24^

The puncture strength test results are depicted in Figure 4c using a 3 mm diameter stainless steel ball according to ASTM F1306‐16 standard at a normal force loading speed of 25 mm min^−1^. In order to demonstrate the superior puncture resistance of our newly prepared P‐class membranes, data on commercial trilayer Celgard 2325 separators (solid lines) are also superposed. Celgard membranes have been well documented to display strong puncture resistances.^25^

The plots in Figure 4c show clearly that the UHMWPE P‐class membranes are much more puncture resistant than that of the commercial Celgard membranes. First, the total load per unit membrane thickness in P‐class membrane is about 52% higher (0.52 vs 0.34 N μm^−1^). Second, it also displays a stronger puncture hardening behavior that is absent from the Celgard membranes. As can be seen from the plots of the first order derivative of puncture load versus penetration displacement in Figure 4c, the P‐class membranes are ≈250% higher in strain hardening rates. Third, the total displacement per membrane thickness of P‐class membrane is also about 250% larger than that of the Celgard separators. The puncture failure displacement per unit membrane thickness in P‐class membrane is ≈0.83 mm μm^−1^ and that for Celgard is ≈0.32 mm μm^−1^. Finally, the mode of failure in the P‐class membrane is localized around the pinhole after fracture, whereas that in the Celgard catastrophic. The Celgard membrane displayed simultaneous film splitting along the machine direction of the membrane accompanied with pinhole fracture. Please see Videos S3 and S4 (Supporting Information) captured during the puncture test on both P‐class and Celgard separators. It should be stated that the total puncture load in the P‐class membrane at 6 μm is ≈3 N which satisfies the safety requirement for separators for use in spiral winding LIBs.^5^

Besides, the puncture hardening versus displacement characteristics suggests that the P‐class membrane separators might help to annihilate lithium dendrites grown during cell charge/discharge processes.^26^ As the reactive load exerted by the separator on the lithium ion dendrite increases faster at large deformations, it is possible that it might reach a value that is comparable with the strength of the dendrites and hence may help to limit dendrite preparation.

It is also worth mentioning that the UHMWPE P‐class membrane also exhibits a strong self‐healing behavior when the applied puncture load is below 100 g, suggesting that the membrane will have high fatigue resistance during the charge/discharge cycles. Complete shape recovery is observed within 2 min after load removal [to be published in a separate publication].

Tensile properties of the D‐class membrane are significantly higher than that of the P‐class at both the ambient temperature and high temperatures. As seen in Figure 4a, the tensile strength of the D‐class membrane is up to 550 MPa. This is 300% stronger than that of the P‐class and about the same as that of a stainless steel! Even at the high temperature of 145 °C, the membrane exhibits a tensile strength of 160 MPa. These high tensile strength values are again due to the shish‐kebab self‐reinforced composite microstructures in the D‐class membrane. The retention of high molecular alignment in conjunction with the densification due to thickness reduction leads to such high tensile strengths. These high tensile strengths are highly desirable for a robust membrane in case of the unfortunate overcharging or runaway reactions in LIBs.

Similar to the tensile performances, the puncture resistance also increased significantly after pore shutdown. As shown in the solid line of Figure S1 (Supporting Information), the maximum puncture load for the D‐class is ≈470 g. It should be noted that the membrane thickness for D‐class is ≈2 μm. This is 4.6 times of that of P‐class membrane in terms of load per unit membrane thickness.

Besides, the dimensional stability during pore closure process is also critical. The strong viscoelasticity of P‐class membrane enables its lateral dimensions to be fully constrained during annealing. As can be seen in Figure S2 (Supporting Information), no lateral dimension shrinkage is observed. The photo Figure S2a (Supporting Information) is the P‐class separator with edges constrained, and the photo Figure S2b (Supporting Information) is the photo immediately after pore closure at 145 °C under constraint. Video S5 (Supporting Information) shows the evolution of membrane morphology as a function of temperature at a heating rate of 10 °C min^−1^. The initial temperature is at 125 °C, and the membrane rapidly transforms into translucent upon the pore closure temperature of 145 °C.

Finally, in order to be an effective thermal fuse to provide the inherent safety protection for LIBs, the separator must exhibit discontinuous impedance increase to shut down the electrical circuit in the event of overcharging runaway reactions. The thermal fuse performances were characterized using electrochemical spectrometry on CHI760E. Nyquist plots for the P‐class (prior to pore closure) and D‐class membranes (after pore closure) sandwiched between blocking stainless steel plate electrodes are depicted in Figure 4d. The porous separators (P‐class) display no semicircle responses at all frequencies, but the dense D‐class separators show a suppressed semicircle at high frequencies followed by a linear increase of *Z*″ against *Z*′ in the low frequency regime. From the intercept of the semicircle arc with *Z*′ axis, the bulk resistance of the D‐class membrane is ≈14 900 Ω.^27^, ^28^ This represents more than 18 000 times increase in comparison that for the P‐class (0.81 Ω). The bulk resistance for P‐class was taken as the intercept of the straight line on *Z*′ axis at high frequencies.^29^ Thus it may be safely assumed that the redox reactions on the battery electrodes will be suppressed and no notable electrical current flow for further temperature rise will take place after pore closure.

**Figure 5:**
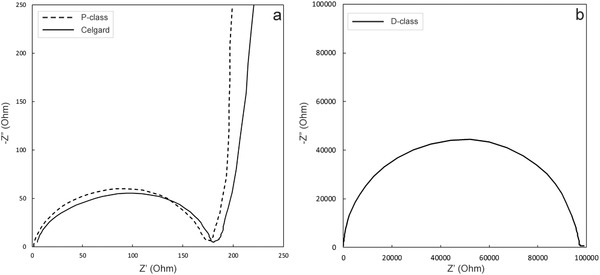
Nyquist plots of a) P‐class and Celgard 2325 trilayer membranes, and b) D‐class membranes assembled in between Li and LiCoO_2._

To further verify these characterizations, EIS measurements on 2025 coin cells were further carried out. Cells were assembled by sandwiching the membrane in between LiCoO_2_/BP2000/PVDF (8:1:1 wt%) cathode and lithium metal anode, with 1 m LiPF_6_ in EC/DMC/EMC (1:1:1 vol%) electrolyte, and after two cycles of activation at 100% discharge state. Nyquist plots for cells prepared using these separators are presented in **Figure** **5**a,b, respectively. The charge transfer resistances are ≈170 Ω for cells prepared using separators prepared with UHMWPE P‐class (dashed line in Figure 5a) and Celgard (solid line in Figure 5a) membranes. Cells prepared using the UHMWPE D‐class membrane shows a more than 550 times increase in charge transfer resistance of 9.7 × 10^4^ Ω.^30^, ^31^ Thus, the pore shutdown is effective in mitigating further electrochemical reactions. Having established the superior safety features of the newly prepared P‐class separators, their applications in cell performance tests are characterized in the next section.

### 2.3 Cell Performance Tests

**Figure 6:**
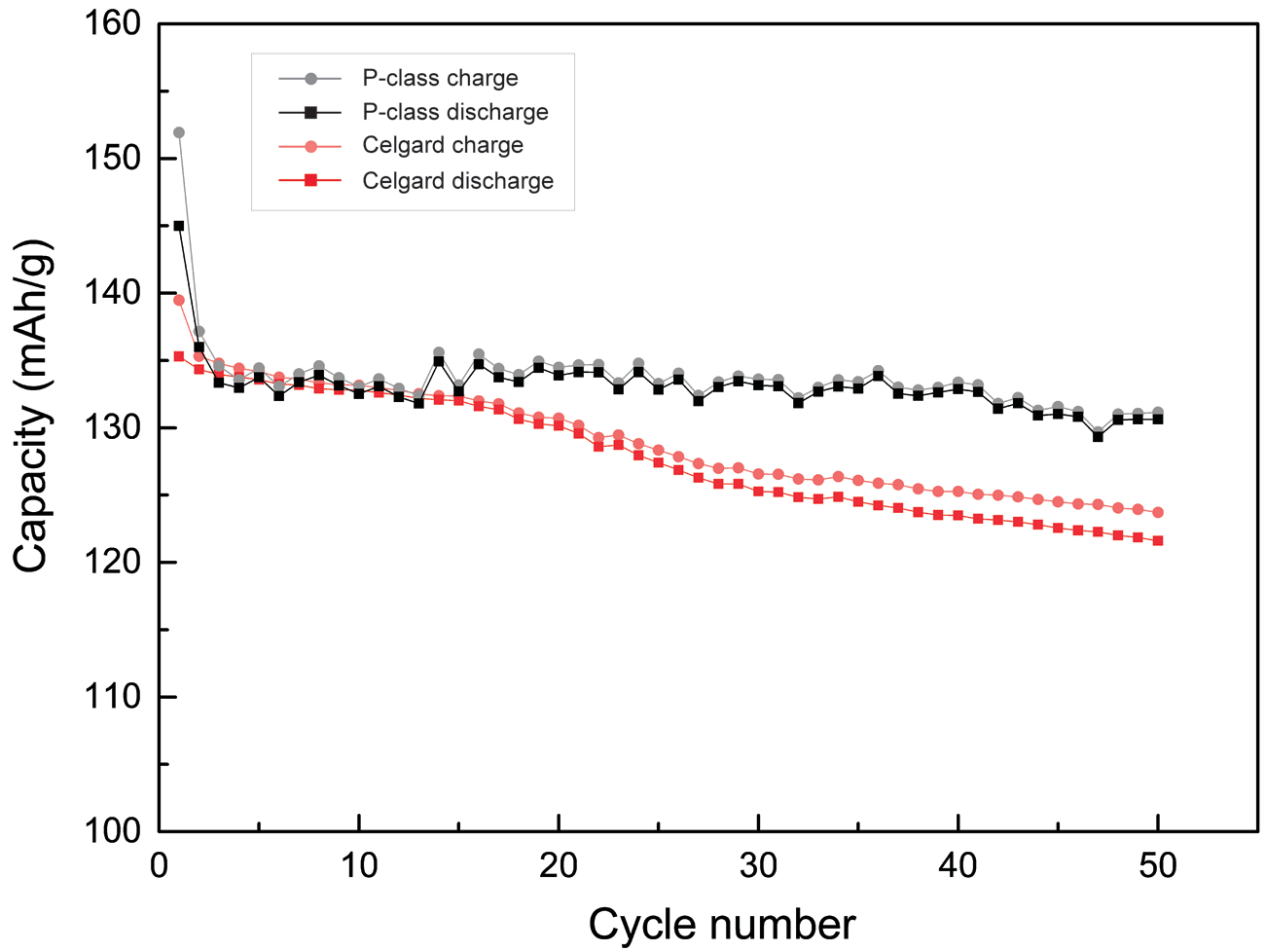
Charge and discharge 1C‐rate capacity of cells utilizing P‐class and Celgard 2325 membranes as separators.

The cyclic cell performance tests at 1 °C charge/discharge rate and 30 °C within the range of 3.0–4.3 V for 50 cycles were carried out on 2025 coin cells prepared using P‐class and Celgard separators as those in Figure 5a. The cyclic dependence capacitance plots are presented in **Figure** **6**. Clearly, the P‐class cells exhibit consistently higher capacity than those of the Celgard especially after 15 cycles. For example, at the end of the 50 cycles, the charge capacitance for P‐class is about 7.4% higher than that of the Celgard (130.6 vs 121.6 mAh g^−1^). The UHMWPE P‐class separator cells also exhibit a more stable charge and discharge capacity compared with Celgard. The capacitance retention by ignoring the first cycle is 96% whereas for Celgard is 90%. It is also important to note that the capacitances measured on charge and discharge at the same cycle for cells prepared using P‐class separators are almost congruent. However, the Celgard cells show increasing hysteresis with the discharge capacitance dropping faster as charge/discharge cycle increases. Hysteresis between charge and discharge in LIBs is believed to be determined by reaction kinetics which will result in fading capacitances. This phenomenon becomes more significant at high charge/discharge rates and efforts in mitigating hysteresis have mostly been concentrated on design of electrodes.^32^, ^33^ The quick charge feature in conjunction with cyclic capacitance retention exhibited by the UHMWPE P‐class separator is extremely desirable in all LIB energy storage devices.^34^

## 3 Conclusion

In summary, the UHMWPE P‐class membrane separators that simultaneously exhibit strong mechanical, electrochemical safety characteristics, and high cyclic cell performance at high charge/discharge rates are successfully synthesized. The high charge rate and cyclic capacity retention efficiency are due to the high volumetric porosity and small membrane thickness. The effective thermal fuse functionality is due to the strong biaxial molecular anisotropy of self‐reinforced composite microstructure. Retention of molecular orientations upon pore closure leads to a drastic 300% increase in tensile and puncture strength. The localized puncture failure mode in the P‐class separator is a result of higher tensile toughness of UHMWPE. Therefore, the newly prepared thin and robust self‐reinforced composite UHMWPE separators with high cycle and cell performances offer solutions to the pareto fronts in current separators.^24^ Additionally, the increasing puncture resistance with displacement suggests that the P‐class membrane separators might potentially mitigate lithium dendrite growths during cell charge/discharge cycles.

## 4 Experimental Section

*UHMWPE Nanoporous Membrane Preparation*: UHMWPE (GUR 4022) was purchased from Celanese, and the average molecular weight was about 3.5 × 10^6^ g mol^−1^. Petrolatum (Protopet 1S) was purchased from Sonneborn, average *M*_w_ = 600 g mol^−1^. 1 wt% antioxidant mixture of Irganox 1010 and Irgafos 168 (1:1 by weight) was used to stabilize the polymer during processing. Then the gel film was freely extruded by HAAKE twin screw extruder with a tape take‐up unit. Then the free extruded gel films were hot stretched twice at 120 °C in two perpendicular directions in sequence on the INSTRON 5567 universal testing system fixed with an environmental chamber. All gel films were drawn sequentially to a total draw ratio of 6 × 6. Dimensional shrinkages were prevented in transverse direction during hot stretching by confining the films laterally during drawing. All films were annealed at 125 °C for 15 min after stretching to relieve internal stresses and induce self‐reinforced composite formation. Petrolatum, the solvent for UHMWPE, was extracted by *n*‐Hexane. Dimensional constraint was applied through all the procedures to introduce membrane porosity after oil removal. Then the highly porous biaxial oriented UHMWPE films were prepared, labeled as P (for “porous”) Class.

*LV‐SEM*: Top‐surfaces of membranes were examined using an FEI Quanta 250 FEG low‐voltage scanning electron microscopic system. The imaging of polymeric samples without any conductive coating was performed at an acceleration voltage of 2 kV and a compensation voltage of 0.8 kV.

*Differential Scanning Calorimetry (DSC)*: Differential scanning calorimetry was carried out on a TA Q1000 DSC, ramping from 0 to 170 °C at 5 °C min^−1^. To simulate the thermal history of annealing with biaxial constraint force, films were folded and loaded between flat aluminum pans, then compressed together before DSC tests. Both on‐set melting point and peak melting point were reported, on‐set melting point was corresponding temperature of intersect of extrapolated baseline and endothermic slope. Relative crystallinity was determined by Equation (2)

(2) $$\chi_{\mathrm{DSC}}=(\Delta H_{m}/\Delta H_{m}^{0})\;\times \;100\%$$

where χ_DSC_ is relative crystallinity based on DSC results, Δ*H_m_* is measured enthalpy of endothermic peaks, and $\Delta H_{m}^{0}$ is the heat fusion of polyethylene, 293.57 J g^−1^.^22^

*Tensile Properties Testing*: ARES‐2000 rheometer (TA Instruments) was used for tensile properties measurement of prepared membranes, taking ASTM D882 as standard reference.

*Puncture Resistance Test*: Puncture resistance test was performed using the similar setup and the same puncture velocity of 25 mm min^−1^ according to the standard ASTM F1306‐16, with a 3 mm diameter stainless steel ball (a little smaller than the standard with diameter of 3.2 mm).

*EIS for Blocking Electrodes Sandwiched Membranes*: 2025 coin cells were assembled with membranes immersed in 1 m LiPF_6_ in EC/DMC/EMC (1:1:1 vol%) electrolyte and sandwiched in between two smooth‐surface stainless steel electrodes. All cells were assembled in an argon‐filled gloved box with water and oxygen less than 0.1 ppm. Before testing the cells were conditioned for 24 h for separators to be fully saturated. AC impedance measurements were carried out using CH760E (CH Instruments) electrochemical workstation over frequency from 0.1 Hz to 100 kHz.

*EIS for Li/LiCoO_2_ Sandwiched Membranes*: 2025 coin cells were assembled with membranes immersed in 1 m LiPF_6_ in EC/DMC/EMC (1:1:1 vol%) electrolyte and sandwiched between LiCoO_2_/BP2000/PVDF (8:1:1 wt%) as the cathode and Li metal as the anode. All cells were assembled in an argon‐filled gloved box with water and oxygen less than 0.1 ppm. AC impedance measurements were performed using CH760E (CH Instruments) electrochemical workstation over frequency from 0.1 to 100 kHz.

*Cell Performance Test*: Charge–discharge test was carried out for the 2025 coin cells assembled by sandwiching the membrane in between LiCoO_2_/BP2000/PVDF (8:1:1 wt%) cathode and lithium metal anode, with 1 m LiPF_6_ in EC/DMC/EMC (1:1:1 vol%) electrolyte. All cells were assembled in an argon‐filled gloved box with water and oxygen less than 0.1 ppm. The cell was charged and discharged at 1 °C and 30 °C within the range of 3.0–4.3 V for 50 cycles using Neware BTS 4000 system.

## Conflict of Interest

The authors declare no conflict of interest.

## Supporting information

Supplementary

Supplementary

Supplementary

Supplementary

Supplementary

Supplementary
