# Supplementary material for: Nanoporous UHMWPE Membrane Separators for Safer and High‐Power‐Density Rechargeable Batteries
Source: Glob Chall. 2017 May 11;1(4):1700020. doi: 10.1002/gch2.201700020 (PMC6607144; doi:10.1002/gch2.201700020)
Supplement: Supplementary file 1 — Supplementary [file GCH2-1-1700020-s001.pdf]

Copyright WILEY-VCH Verlag GmbH & Co. KGaA, 69469 Weinheim, Germany, 2017.

Supporting Information

for *Global Challenges,* DOI: 10.1002/gch2.201700020

Nanoporous UHMWPE Membrane Separators for Safer and High-Power-Density Rechargeable Batteries

*Runlai Li and Ping Gao**

Supporting Information

Nanoporous UHMWPE Membrane Separators for Safer and High-Power Density Rechargeable Batteries

*Runlai LI and Ping GAO**

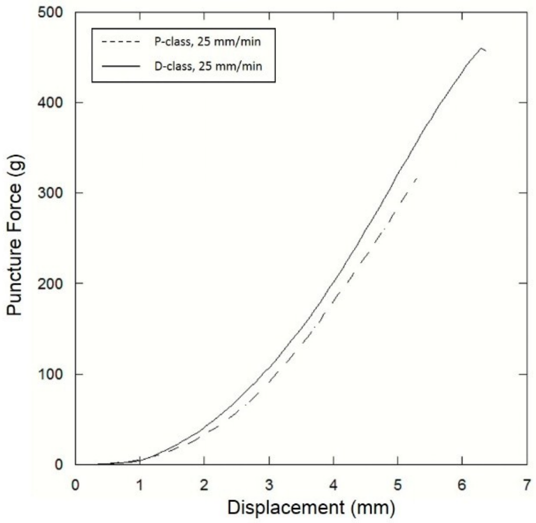

Figure S1 Force-penetration displacement curves of P-class and D-class for pucnture resistance test.

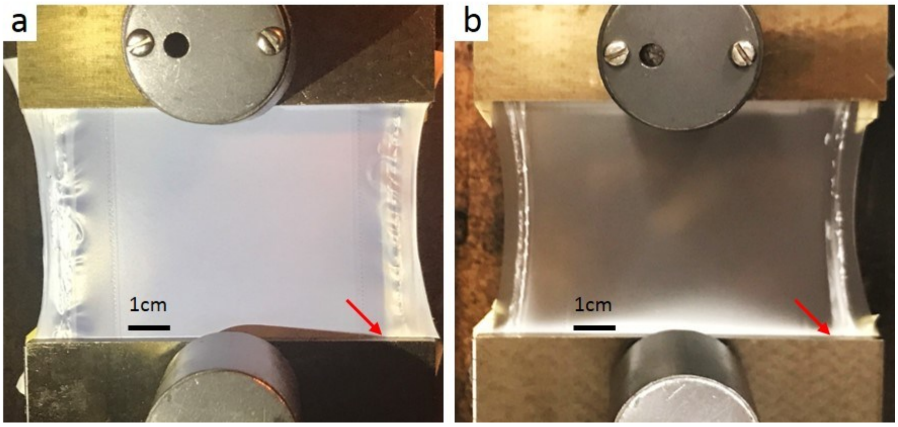

Figure S2 Photos of P-class membrane before and after pore closure. (a) at room temperature. (b) immediately after pore closure at 145 ^{o}C. The red arrows indicate the edge of the membrane separator before and after pore closure.
